# Burst versus continuous delivery design in digital mental health interventions: Evidence from a randomized clinical trial

**DOI:** 10.1177/20552076241249267

**Published:** 2024-04-30

**Authors:** Marta Anna Marciniak, Lilly Shanahan, Kenneth S L Yuen, Ilya Milos Veer, Henrik Walter, Oliver Tuescher, Dorota Kobylińska, Raffael Kalisch, Erno Hermans, Harald Binder, Birgit Kleim

**Affiliations:** 1 Department of Psychology, 27217 University of Zurich, Zurich, Switzerland; 2 Department of Psychiatry, Psychotherapy and Psychosomatics, Psychiatric University Hospital (PUK), 27217 University of Zurich, Zurich, Switzerland; 3 Jacobs Center for Productive Youth Development, 27217 University of Zurich, Zurich, Switzerland; 4 539160 Leibniz Institute for Resilience Research (LIR), Mainz, Germany; 5 Neuroimaging Center (NIC), Focus Program Translational Neuroscience (FTN), 9182 Johannes Gutenberg University Medical Center, Mainz, Germany; 6 Department of Developmental Psychology, 1234 University of Amsterdam, Amsterdam, The Netherlands; Research Division of Mind and Brain, Department of Psychiatry and Psychotherapy CCM; 7 14903 Charité - Universitätsmedizin Berlin, Corporate Member of Freie Universität Berlin, Humboldt-Universität zu Berlin, and Berlin Institute of Health, Berlin, Germany; 8 Institute for Molecular Biology (IMB), Mainz, Germany; 9 Institute for Psychology, 49605 University of Warsaw, Warsaw, Poland; 10 Radboud University Medical Center, 198328 Donders Institute for Brain, Cognition, and Behaviour, Nijmegen, The Netherlands; 11 Institute of Medical Biometry and Statistics, Faculty of Medicine and Medical Center, 9174 University of Freiburg, Freiburg, Germany; 12 Freiburg Center for Data Analysis and Modelling, 9174 University of Freiburg, Freiburg, Germany

**Keywords:** Ecological momentary intervention, digital health, mental health, intervention delivery, burst delivery design, reappraisal

## Abstract

**Objective:**

Digital mental health interventions delivered via smartphone-based apps effectively treat various conditions; however, optimizing their efficacy while minimizing participant burden remains a key challenge. In this study, we investigated the potential benefits of a burst delivery design (i.e. interventions delivered only in pre-defined time intervals) in comparison to the continuous delivery of interventions.

**Methods:**

We randomly assigned 93 participants to the continuous delivery (CD) or burst delivery (BD) group. The CD group engaged in ReApp, a mobile app that increases positive cognitive reappraisal with a consistent delivery schedule that provides five prompts per day throughout the 3-week-long study, while the BD group received five daily prompts only in the first and third weeks of the study.

**Results:**

No significant differences were found between the groups in terms of adherence, mental health outcomes (specifically depressive and anxiety symptoms), level of perceived stress, and perceived helpfulness of intervention. The BD group showed a significantly decreased perceived difficulty of intervention over time.

**Conclusions:**

The results suggest that the burst delivery may be as suitable for digital mental health interventions as the continuous delivery. The perceived difficulty of the intervention declined more steeply for the BD group, indicating that it improved the feasibility of the positive cognitive reappraisal intervention without hurting its efficacy. This outcome may inform the design of less burdensome interventions with improved outcomes in future research.

## Introduction

Digital mental health interventions delivered via smartphones are widely used in both preventative and intervention contexts,^1–4^ demonstrating effectiveness both as standalone treatments^5^ and blended therapies.^6^ They are usually administrated as ecological momentary interventions (EMIs), that is, treatments delivered to a person in real-life settings,^7^ and typically tested in randomized trial designs. While they are readily available for dissemination, cost-effective and easily accessible,^8^ the factors contributing to their effectiveness have not yet been widely investigated.^8,9^

Previous studies identified the components of cognitive–behavioral therapy (CBT), gamification, personalization, and social relationships as potential factors enhancing the efficacy and acceptability of EMIs^10–12^. However, the impact of the EMI delivery design remains largely unexplored. Existing reviews and meta-analyses on EMI research have focused on the continuous delivery of interventions, wherein participants use the app consistently throughout the whole study^2,5,13–16^ in contrast to the burst design, wherein the engagement of participants is only in pre-defined time intervals. The same can be said for Just-in-Time Adaptive Interventions (JITAIs), which are EMIs adapting over time to a person's changing internal and contextual state.^17^ JITAIs are typically tested in adjustable and changing-over-time study designs^18^ (e.g. in terms of the active components of the intervention or the frequency of the intervention). However, to the best of our knowledge, there have been no mental health-oriented JITAIs tested with various delivery designs. Thus, there is no empirical evidence that the continuous delivery design is actually the most effective in reducing mental health symptoms, increasing adherence, or enhancing participant engagement.

At the same time, the burst delivery design has been a longstanding practice in ecological momentary assessment (EMA) studies utilized to capture the dynamics of psychological processes.^19^ The application of the burst delivery design in EMIs holds promise for addressing a key challenge in the mobile health (mHealth) field, namely, the reduction of participants' burden and increase of their engagement, especially in trials with extended intervention periods lasting several months. Indeed, new EMI study aims to employ the burst delivery design, such as a 9-month design with intervention bursts every 3 weeks, alternating with “quiet” periods, during which participants use the intervention at their own discretion without being reminded about its usage.^20^ Potentially, the burst delivery design could also increase the effectiveness of EMIs. Previous research suggested that interventions are most effective when participants apply previously learned cognitive strategies at moments when they are needed most.^21^ However, such studies have so far not been conducted under digital mental health settings.

The current study aims to investigate the differences between two key delivery design features, both within- and between-conditions, namely, continuous and burst delivery designs of digital mental health interventions with regard to the following outcomes:

- adherence to the EMI;
- changes in the perceived helpfulness and difficulty of the intervention throughout the study;
- changes in the mental health indices (specifically depressive and anxiety symptoms) and perceived stress in baseline versus follow-up assessments; and
- changes in the target engagement involving changes in the tendency to use the CBT component included in the EMI in baseline versus follow-up assessments.

To investigate the abovementioned differences, we implemented an EMI mobile app, called ReApp, for this randomized clinical trial.^22^ ReApp is solely based on the therapeutic component of a positive cognitive reappraisal (PCR), which eliminates the confounding factors associated with using multiple therapeutic targets and strategies within one EMI. Moreover, PCR is a well-researched and central CBT component^23,24^ and a core resilience factor^25,26^ encouraging the users to find positive reinterpretations to events appraised by them as stressful or negative.^27^ Thus, the findings pertaining to this therapeutic target can hold high relevance for both clinical and preventative practices.

## Methods

### Study design

This study was a three-armed randomized controlled trial but the current manuscript focuses on two arms involving 93 participants who received intervention and were randomly allocated to either the continuous delivery (CD) or burst delivery (BD) design group. The third arm, which represents the control condition, did not receive an intervention and is not included in the current work. Please see Marciniak et al.^22^ The study proposal received approval from the Ethics Committee for the Faculty of Arts and Social Sciences at the University of Zurich (approval #21.2.12). The ClinicalTrials.gov identifier was NCT05784831. We followed the CONSORT guidelines while preparing the manuscript.

### Ecological momentary intervention

ReApp, the intervention used in this study, comprised two components: EMA and EMI. EMA was employed to assess the participants’ daily mood and adapted from Vaessen et al. and Wackerhagen et al.^28,29^ The participants rated short sentences, such as “I feel sad” and “I feel peaceful,” on a scale from 1 (not at all) to 7 (very much). EMA was delivered five times per day at pseudo-random 3-hour time windows from 8:00 AM to 10:30 PM. For three times daily, EMA was combined with the EMI component, with participants recalling a stressful event and generating three positive reappraisals for this event.

### Measures

**Figure 1.**
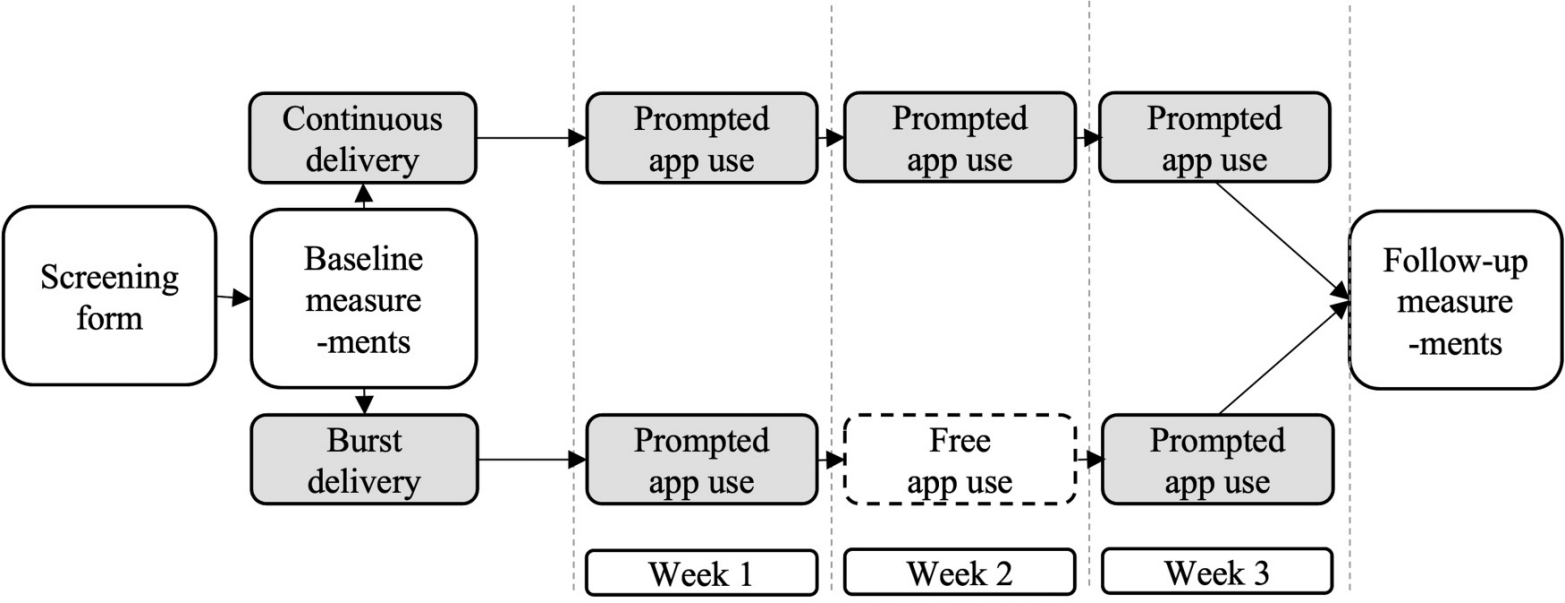
Study design.

We calculated the adherence (i.e. the number of completed surveys by each participant) to investigate the differences between the groups in terms of engagement with ReApp. To investigate the perceived helpfulness and difficulty of the EMI, we extracted two questions from the EMI protocol, namely, “I feel better after reappraising this experience,” and “It was hard for me to reappraise this experience,” which were rated on a scale from 1 (not at all) to 7 (very much) after each PCR exercise performed within the app in the 21 days of the study duration. To compare the effects of the EMI on mental health, we assessed the depressive symptoms, anxiety and perceived stress at the baseline and follow-up meetings (Figure 1) using the German translations of the Beck Depression Inventory-II (BDI-II),^30^ the State–Trait Anxiety Inventory (STAI)^31^ and the Perceived Stress Scale (PSS).^32^ The self-reported tendency to use PCR was indexed by the Positive Reappraisal subscale of the Cognitive Emotion Regulation Questionnaire (CERQ)^33^ and the Cognitive Reappraisal scale of the Emotion Regulation Questionnaire (ERQ).^27^

### Study procedure

The enrolment of participants began in April 2021 and concluded in May 2022. Individuals interested in the study completed a short online screening form. The inclusion criteria were as follows: (1) being a student of a higher education institution; (2) being 18–29 years old; (3) having sufficient knowledge of the German language; (3) being a smartphone user; and (4) obtaining 12 points or less on a 20-point Positive Reappraisal subscale of CERQ. This cutoff score was set based on the mean score obtained from the sample mirroring the target population (i.e. healthy Swiss students of a higher education institution). The exclusion criteria included self-reported mental illness or ongoing psychotherapy. All participants provided written informed consent. Individuals who fit the inclusion criteria were eligible to participate in the study. Randomization into the groups was conducted with the use of the n = 3 block algorithm generated by an independent researcher, meaning that three participants were grouped together for randomization to the study conditions. At the baseline meeting, which was conducted online due to restrictions related to the COVID-19 pandemic, the participants got familiarized with the definition and examples of PCR and were instructed on how to use the EMI, including the information that they can self-initiate it whenever they feel they can benefit from it. After an online baseline meeting, the participants used ReApp for 3 weeks, with the CD group participants receiving five prompts reminding them to use ReApp every day. The BD participants did not receive these prompts in the second week of the study, but were encouraged to use the app freely during this time depending on their needs. After 3 weeks, the participants completed follow-up questionnaires (Figure 1). They received charts showing their mood fluctuations during the period of app usage and were remunerated up to 105 Swiss francs or six university credit points to compensate for their time.

### Analysis

All analyses were performed in R (version 4.0.4) using R Studio (version 1.4.1).

The sample size calculation was conducted with the use of the sjstats package.^34^ There were no publications comparing the burst and continuous delivery designs; hence, we estimated the expected effect size based on the previous work on the effects of EMIs on the mental health indices comparing the intervention effects to the active control conditions.^5,16^ Hence, for the effect size of d = 0.6, alpha of 0.05, and power of 80%, the required sample size was 94 participants. However, due to a prolonged recruitment process, we stopped the recruitment at 93 participants.

Adherence was calculated as the sum of completed surveys. Additionally, we separately indexed the number of completed automatically-delivered surveys and the number of user-initiated surveys for each condition. The statistical difference in adherence was evaluated with Student's *t*-tests. The EMI data were nested within each day (21 days), further within participant (93 participants), and two conditions. The missing data were imputed with the k-nearest neighbor method (i.e. mean of the neighbors nearest to the missing value).^35,36^ With this method, we imputed 9% of the EMI data and none of the questionnaire data. For both EMI data and questionnaires, we performed a linear mixed-model (LMM) analysis with the nlme package.^37^ Group and Time were included in the models as fixed effects and interaction terms. We also separately assessed the effect of Time for both groups. We then calculated Nakagawa's marginal R2 *m* values for the LMM (variance explained by fixed effects) and included these numbers in Supplement 1. When needed, data transformation was performed as described in the Results section. The selection of the most suitable transformation method was based on the visual inspection of data.

## Results

### Sample

**Figure 2.**
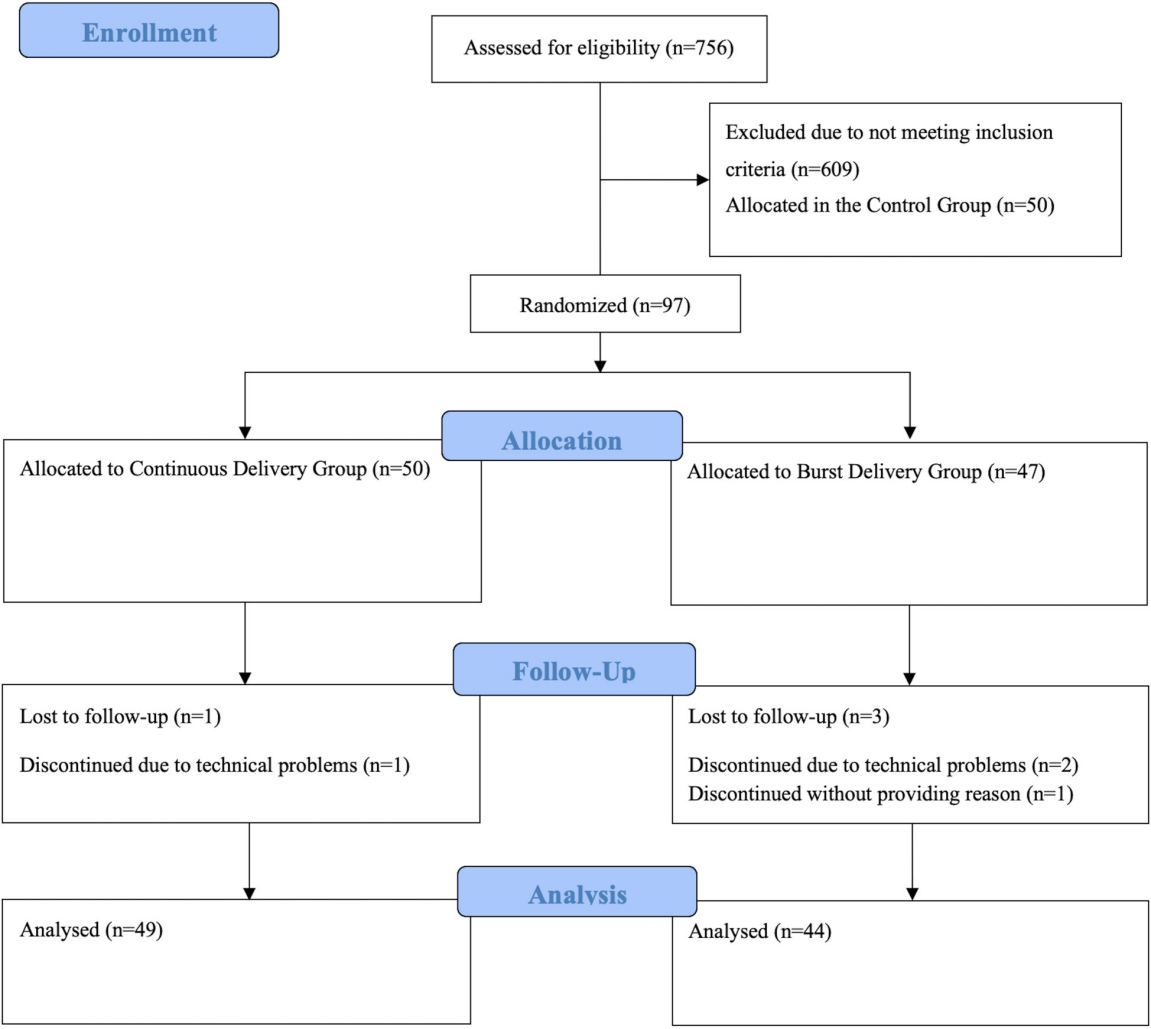
CONSORT flow diagram.

Out of 756 individuals who completed the online screening, 147 were eligible to participate in the study and randomly assigned to one of the three groups. Fifty participants were assigned to the control condition. They did not receive intervention, only a self-monitoring tool. These participants were not included in the analyses of the current manuscript. Please see Marciniak et al. for details.^22^ Three participants dropped out due to technical problems (i.e. the app did not work on their phones). One dropped out without providing a reason. Ninety-three participants from the BD and CD groups completed the procedure (Figure 2).

Of the 93 participants, 89 were female, and four were male. No participants identified as another gender. The mean age of the participants was 22.01 years, SD = 2.42, and range 18–29. All participants were German-speaking students from higher education institutions. No significant differences in these characteristics between the groups were found at baseline (Table 1).

**Table 1.** Demographic characteristics of the sample.

	Continuous delivery	Burst delivery	Statistical difference
Mean age [SD]	22.02 [2.55]	22.00 [2.30]	*t* = −0.04, *p* = 0.968
Percentage of students	100%	100%	–
Gender ratio Female/Male/Diverse	47/2/0 95.9% female	42/2/0 95.4% female	*t* = 0.109, *p* = 0.914
Mean baseline score in PSS [SD]	21.80 [6.82]	22.46 [6.69]	*t* = 0.50, *p* = 0.640
Mean baseline score in STAI [SD]	43.61 [9.39]	41.30 [9.41]	*t* = -1.19, *p* = 0.238
Mean baseline score in BDI-II [SD]	13.59 [8.38]	13.96 [9.00]	*t* = 0.20, *p* = 0.841
Mean baseline score in CERQ [SD]	9.51 [2.04]	9.09 [2.03]	*t* = −0.99, *p* = 0.324
Mean baseline score in ERQ [SD]	21.46 [4.55]	19.55 [4.89]	*t* = −1.95, *p* = 0.054
Mean follow-up score in PSS [SD]	18.65 [5.79]	18.64 [5.81]	Tables 3, 4 and 5
Mean follow-up score in STAI [SD]	41.06 [8.97]	40.14 [8.47]	Tables 3, 4 and 5
Mean follow-up score in BDI-II [SD]	10.04 [7.86]	8.87 [7.82]	Tables 3, 4 and 5
Mean follow-up score in CERQ [SD]	12.84 [3.30]	12.30 [3.30]	Tables 3, 4 and 5
Mean follow-up score in ERQ [SD]	23.35 [4.07]	20.17 [5.04]	Tables 3, 4 and 5

### Adherence

Each participant from the CD group received a total of 105 prompts, of which 63 included the EMI component. Each participant from the BD group received 70 prompts, of which 42 included the EMI component. Both groups had the option to initiate more EMIs whenever they felt they could benefit from them.

As expected, there was a significant difference (*t* = −2.86, *p* = 0.005) in completing the automatically delivered surveys, with the CD group filling in more surveys (*m* = 49.22, SD = 24.82, 47% of planned surveys vs. *m* = 35.09, SD = 19.86, 50% of planned surveys in BD) as they were prompted for seven more days. On average, the BD group participants self-initiated more surveys (*m* = 40.16, SD = 25.98) when compared to the CD group ones (*m* = 35.74, SD = 29.38), but this difference was not significant (*t* = 0.59, *p* = 0.555). Overall, the CD participants on average completed 84.96 surveys (81% of the surveys planned for this group), while the BD participants completed 75.25 surveys (104% of the planned surveys for this group). The difference between the groups in the overall adherence (i.e. both prompted and self-initiated surveys) was not significant (*t* = −1.65, *p* = 0.103) (Table 2).

**Table 2.** Adherence rates.

	Continuous delivery	Burst delivery	Statistical difference
Completed automatically delivered surveys [SD]	49.22 [24.82] (out of 105)	35.09 [19.86] (out of 70)	*t* = −2.86, *p* = 0.005
Completed user-initiated surveys [SD]	35.74 [29.38]	40.16 [25.98]	*t* = 0.59, *p* = 0.555
All completed surveys [SD]	84.96 [30.69]	75.25 [31.92]	*t* = −1.65, *p* = 0.103

### Perceived helpfulness of intervention

**Figure 3.**
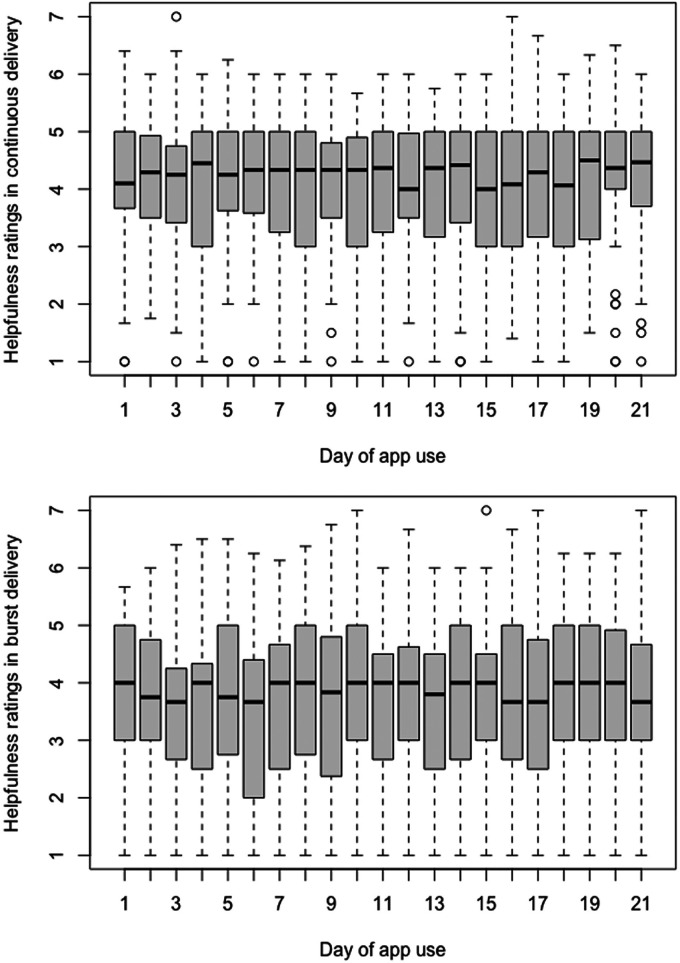
Perceived helpfulness of intervention.

The intraclass correlation coefficient was significant ICC(1) = 0.59, *p* < 0.001, ICC(2) = 0.97. There were no significant effects of Time on either the CD or BD group (i.e. β = −0.002, *p* = 0.503, Cohen's *f* = 0.02, 90% CI [0.00, 0.07] and β = 0.003, p = 0.590, Cohen's *f* = 0.02, 90% CI [0.00, 0.07], respectively), meaning that both groups showed no differences in the perceived helpfulness of the EMI over the course of the study. However, the CD participants consistently assessed the intervention as more helpful, irrespective of the study time (i.e. β = −0.506, *p* = 0.031, Cohen's *f* = 0.21, 90% CI [0.02, 0.39]) (Tables 3, 4 and 5 and Figure 3). The Group × Time (21 days) interaction was not significant (i.e. β = −0.056, *p* = 0.340, Cohen's *f* = 0.02, 90% CI [0.00, 0.06]) and did not explain more variance compared to the fixed effect of Group (R2*m* = 0.026 for both Group × Time interaction and Group, see Supplement 1).

**Table 3.** Results of interaction analyses.

Outcome	Value (β)	SE	DF	*t*-Value	*p*-Value	Cohen's *f* [90% CI]
Perceived helpfulness						
Intercept	3.626	0.17	1858	21.72	<0.001	
Time	0.003	0.00	1858	0.55	0.581	0.00 [0.00, 0.02]
Group	0.506	0.23	91	2.20	0.031	0.21 [0.02, 0.39]
Time × Group	−0.056	0.00	1858	−0.85	0.340	0.02 [0.00, 0.06]
Perceived difficulty						
Intercept	1.191	0.04	1858	44.74	<0.001	
Time	−0.006	0.00	1858	−4.19	<0.001	0.05 [0.01, 0.09]
Group	−0.067	0.06	91	−1.14	0.258	0.04 [0.00, 0.19]
Time × Group	0.007	0.00	1858	3.61	0.001	0.08 [0.05, 0.12]
Perceived stress (PSS)						
Intercept	4.680	0.11	91	43.35	<0.001	
Time	−0.414	0.14	91	−3.02	0.003	0.42 [0.24, 0.60]
Group	−0.073	0.15	91	−0.49	0.623	0.03 [0.00, 0.19]
Time × Group	0.075	0.19	91	0.40	0.692	0.04 [0.00, 0.21]
Anxiety symptoms (STAI)						
Intercept	5.33	0.05	91	117.34	<0.001	
Time	−0.037	0.04	91	−0.83	0.411	0.21 [0.02, 0.39]
Group	0.080	0.06	91	1.28	0.205	0.11 [0.00, 0.28]
Time × Group	−0.048	0.06	91	−0.78	0.440	0.08 [0.00, 0.25]
Depressive symptoms (BDI-II)						
Intercept	3.539	0.20	91	18.00	<0.001	
Time	−0.866	0.19	91	−4.60	<0.001	0.59 [0.40, 0.78]
Group	−0.074	0.27	91	−0.27	0.786	0.02 [0.00, 0.17]
Time × Group	0.256	0.26	91	0.99	0.325	0.10 [0.00, 0.28]
Reappraisal (CERQ)						
Intercept	2.996	0.06	91	48.20	<0.001	
Time	0.479	0.07	91	6.53	<0.001	1.01 [0.79, 1.22]
Group	0.069	0.09	91	0.80	0.424	0.11 [0.00, 0.28]
Time × Group	0.010	0.10	91	0.10	0.920	0.01 [0.00, 0.10]
Reappraisal (ERQ)						
Intercept	19.549	0.70	91	27.98	<0.001	
Time	0.621	0.78	91	0.80	0.427	0.25 [0.08, 0.43]
Group	1.913	0.96	91	1.99	0.050	0.33 [0.16, 0.51]
Time × Group	1.267	1.07	91	1.18	0.241	0.12 [0.00, 0.30]

**Table 4.** Main effect of time for continuous delivery.

Outcome	Value (β)	SE	DF	*t*-Value	*p*-Value	Cohen's *f* [90% CI]
Perceived helpfulness						0.02 [0.00, 0.07]
Intercept	4.131	0.14	958	30.38	<0.001	
Time	−0.002	0.00	958	−0.67	0.503	
Perceived difficulty						0.03 [0.00, 0.08]
Intercept	1.851	0.04	958	47.37	<0.001	
Time	0.001	0.00	958	0.95	0.343	
Perceived stress (PSS)						0.36 [0.11, 0.61]
Intercept	4.607	0.11	48	44.81	<0.001	
Time	−0.339	0.13	48	−2.51	0.015	
Anxiety symptoms (STAI)						0.39 [0.14, 0.64]
Intercept	5.414	0.04	48	125.74	<0.001	
Time	−0.085	0.03	48	−2.71	0.009	
Depressive symptoms (BDI-II)						0.51 [0.25, 0.76]
Intercept	3.465	0.19	48	18.23	<0.001	
Time	−0.609	0.17	48	−3.51	0.001	
Reappraisal (CERQ)						0.97 [0.68, 1.26]
Intercept	3.064	0.06	48	52.46	<0.001	
Time	0.489	0.07	48	6.75	<0.001	
Reappraisal (ERQ)						0.37 [0.12, 0.61]
Intercept	21.462	0.62	48	34.80	<0.001	
Time	1.888	0.73	48	2.57	0.013	

**Table 5.** Main effect of time for burst delivery.

Outcome	Value (β)	SE	DF	*t*-Value	*p*-Value	Cohen's *f* [90% CI]
Perceived helpfulness						0.02 [0.00, 0.07]
Intercept	3.626	0.19	900	19.06	<0.001	
Time	0.003	0.00	900	0.54	0.590	
Perceived difficulty						0.13 [0.07, 0.18]
Intercept	1.919	0.04	900	42.75	<0.001	
Time	−0.007	0.00	900	−3.86	<0.001	
Perceived stress (PSS)						0.51 [0.25, 0.78]
Intercept	22.455	0.94	43	23.77	<0.001	
Time	−3.818	1.13	43	−3.38	0.002	
Anxiety symptoms (STAI)						0.10 [0.00, 0.35]
Intercept	5.334	0.05	43	117.28	<0.001	
Time	−0.037	0.06	43	−0.67	0.505	
Depressive symptoms (BDI-II)						0.68 [0.40, 0.96]
Intercept	3.539	0.19	43	18.43	<0.001	
Time	−0.866	0.19	43	−4.47	<0.001	
Reappraisal (CERQ)						1.03 [0.71, 1.33]
Intercept	2.181	0.04	43	55.54	<0.001	
Time	0.291	0.04	43	6.72	<0.001	
Reappraisal (ERQ)						0.12 [0.00, 0.37]
Intercept	19.550	0.75	43	26.11	<0.001	
Time	0.621	0.78	43	0.79	0.432	

### Perceived difficulty of intervention

**Figure 4.**
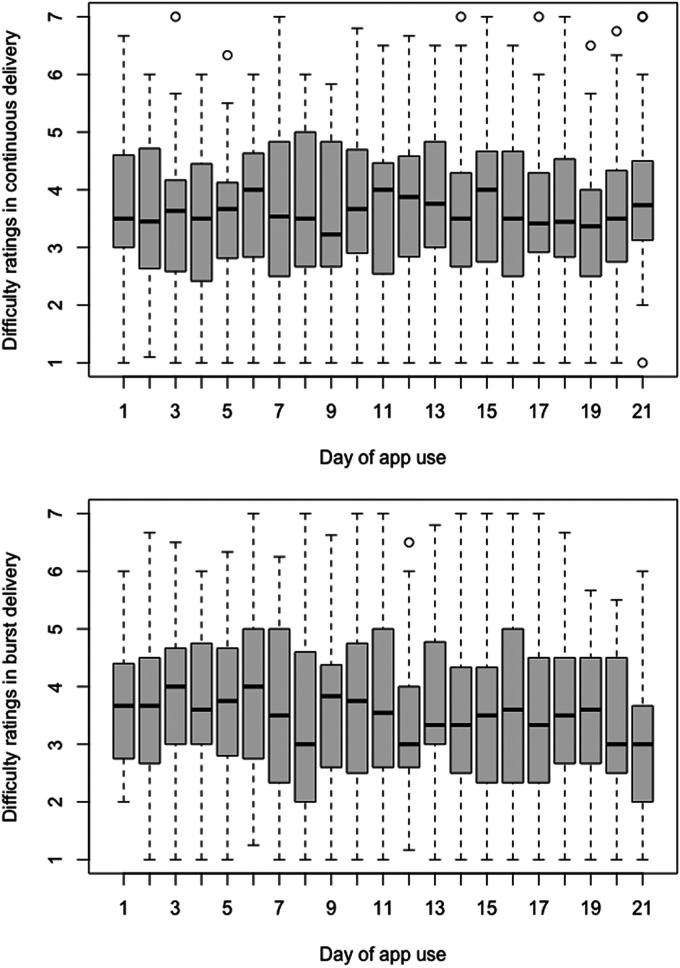
Perceived difficulty of intervention.

The intraclass correlation coefficient was significant ICC(1) = 0.41, *p* < 0.001, ICC(2) = 0.94. Due to the non-normal distribution of residuals, a data transformation was performed, and visual inspection revealed that the best fit was the model with the square root-transformed data. The Group × Time (21 days) interaction was significant (i.e. β = 0.007, *p* = 0.001, Cohen's *f* = 0.08, 90% CI [0.05, 0.12]), and there was a significant effect of Time in the BD group (i.e. β = −0.007, *p* < 0.001, Cohen's *f* = 0.13, 90% CI [0.07, 0.18]), but not in the CD group (i.e. β = 0.001, *p* = 0.343, Cohen's *f* = 0.03, 90% CI [0.00, 0.08]), indicating that the perceived difficulty of the intervention significantly decreased only in the BD group, as shown in Tables 3, 4 and 5 and Figure 4).

### Correlations between perceived helpfulness and difficulty of the intervention

There was a modest, but statistically significant negative correlation found between helpfulness and difficulty of the EMI in the full sample (i.e. r = −0.15, *p* < 0.001, 95% CI [−0.19, −0.11], *t*(1951) = −6.67). This correlation was, however, more negative in the BD group (i.e. r = −0.22, *p* < 0.001, 95% CI [−0.28, −0.16], *t*(943) = −7.06) than in the CD group (i.e. r = −0.06, *p* < 0.05, 95% CI [−0.12, 0.00], *t*(1006) = −1.99). These results suggest that the reappraisals which were easier for the participants to generate, were also perceived as somewhat more helpful to them, especially in the BD group.

### Perceived stress scale

**Figure 5.**
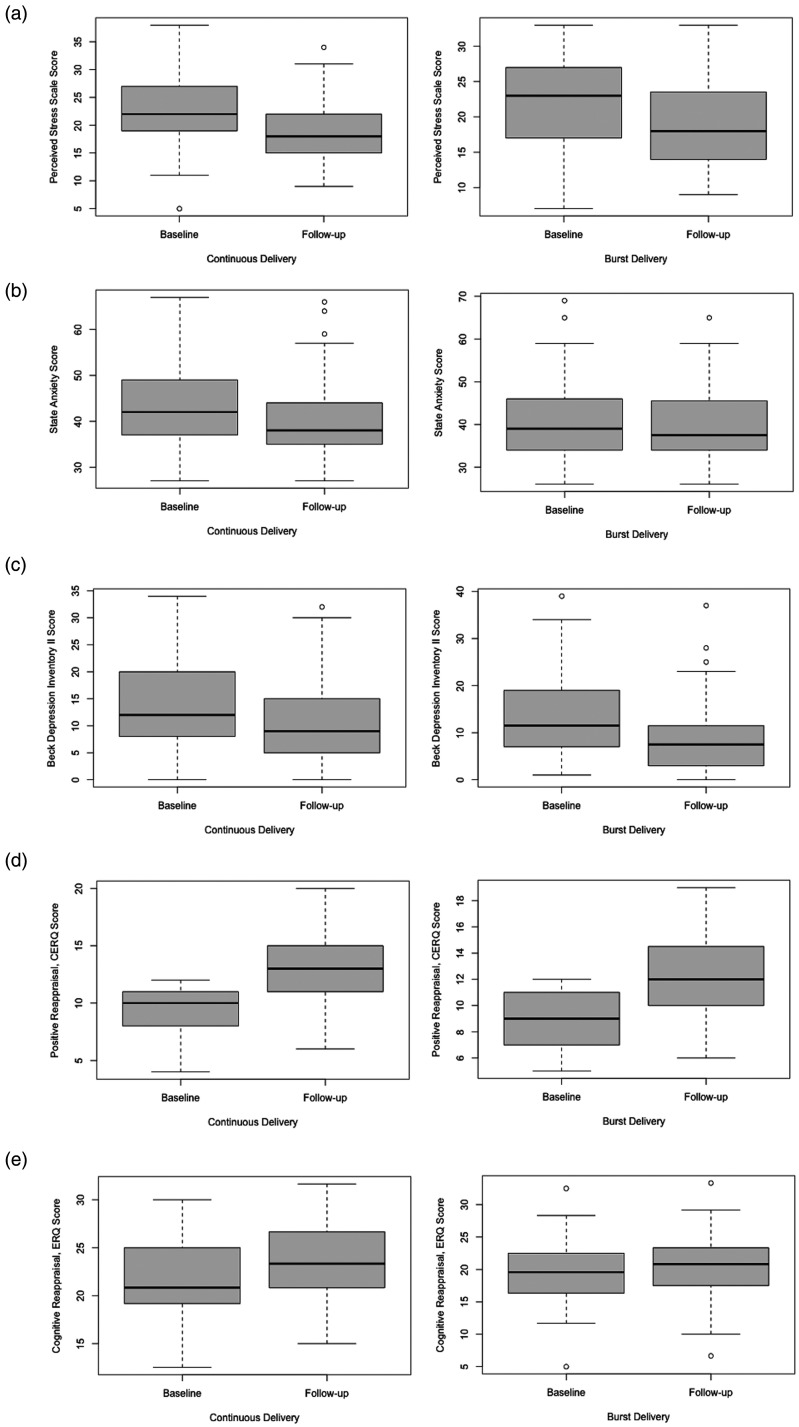
Mental health outcomes of intervention.

The intraclass correlation coefficient was not significant ICC(1) = 0.13, *p* = 0.105, ICC(2) = 023, indicating a low heterogeneity of the scores. Due to the non-normal distribution of residuals, a data transformation was performed, and visual inspection revealed that the best fit was the model with the square root-transformed data. The Group × Time (baseline vs. follow-up) interaction was not significant (i.e. β = 0.075, *p* = 0.692, Cohen's *f* = 0.04, 90% CI [0.00, 0.21]) and did not explain more variance than the fixed effect of time (R2*m* = 0.065 for both, see Supplement 1). There was a significant effect of Time in both the CD and BD groups (i.e. β = −0.339, *p* = 0.015, Cohen's *f* = 0.36, 90% CI [0.11, 0.61] and β = −3.818, *p* = 0.002, Cohen's *f* = 0.51, 90% CI [0.25, 0.78], respectively). This result indicates that both groups decreased their level of perceived stress, as shown in Tables 3, 4 and 5 and Figure 5A.

### State–Trait Anxiety Inventory

The intraclass correlation coefficient was significant (i.e. ICC(1) = 0.51, *p* < 0.001, ICC(2) = 067). Due to the non-normal distribution of residuals, a data transformation was performed, and visual inspection revealed that the best fit was the model with the log-transformed data. The Group × Time (baseline vs. follow-up) interaction was not significant (i.e. β = −0.048, *p* = 0.440, Cohen's *f* = 0.08, 90% CI [0.00, 0.25]). However, there was a significant effect of Time on the CD group (i.e. β = −0.085, *p* = 0.009, Cohen's *f* = 0.39, 90% CI [0.14, 0.64]), indicating a decrease in the anxiety symptoms over the course of the study. In the BD group, the effect of Time was not significant (i.e. β = −0.037, *p* = 0.505, Cohen's *f* = 0.10, 90% CI [0.00, 0.35]), as shown in Tables 3, 4 and 5 and Figure 5B.

### Beck Depression Inventory II

The intraclass correlation coefficient was significant (i.e. ICC(1) = 0.40, *p* < 0.001, ICC(2) = 0.57). Due to the non-normal distribution of residuals, a data transformation was performed, and visual inspection revealed that the best fit was the model with the square root-transformed data. The Group × Time (baseline vs. follow-up) interaction was not significant (i.e. β = 0.256, *p* = 0.325, Cohen's *f* = 0.10, 90% CI [0.00, 0.28]). There was a significant effect of Time on both the CD and BD groups (i.e. β = −0.609, *p* < 0.001, Cohen's *f* = 0.51, 90% CI [0.25, 0.76] and β = −0.866, *p* < 0.001, Cohen's *f* = 0.68, 90% CI [0.40, 0.96], respectively), meaning that both groups experienced a decrease in depressive symptoms over the course of the study, as shown in Tables 3, 4 and 5 and Figure 5C.

### Positive Reappraisal scale, Cognitive Emotion Regulation Questionnaire

The intraclass correlation coefficient was not significant (i.e. ICC(1) = 0.04, *p* = 0.658, ICC(2) = −0.08), indicating a low heterogeneity of the scores. Due to the non-normal distribution of residuals, a data transformation was performed, and visual inspection revealed that the best fit was the model with the square root-transformed data. The Group × Time (baseline vs. follow-up) interaction was not significant (i.e. β = 0.010, *p* = 0.920 Cohen's *f* = 0.01, 90% CI [0.00, 0.10]). There was a significant effect of Time in both the CD and BD groups (i.e. β = 0.489, *p* < 0.001, Cohen's *f* = 0.97, 90% CI [0.68, 1.26] and β = 0.291, *p* < 0.001, Cohen's *f* = 1.03, 90% CI [0.71, 1.33], respectively), meaning that both groups experienced an increase in the tendency to use PCR over the course of the study, as shown in Tables 3, 4 and 5 and Figure 5D.

### Cognitive Reappraisal scale, Emotion Regulation Questionnaire

The intraclass correlation coefficient was significant (i.e. ICC(1) = 0.40, *p* < 0.001, ICC(2) = 0.57). The Group × Time (baseline vs. follow-up) interaction was again not significant (i.e. β = 1.267, *p* = 0.241, Cohen's *f* = 0.12, 90% CI [0.00, 0.30]). There was a significant effect of time on the CD group (i.e. β = 1.888, *p* = 0.013, Cohen's *f* = 0.37, 90% CI [0.12, 0.61]), indicating an increase in the tendency to use reappraisal. By contrast, it had no significant effect on the BD group (i.e. β = 0.621, p = 0.432, Cohen's *f* = 0.12, 90% CI [0.00, 0.37]), as presented in Tables 3, 4 and 5 and Figure 5E.

## Discussion

In this study, we investigated the differences in the effectiveness of continuous versus burst delivery design of digital mental health. To the best of our knowledge, this is one of the first studies offering a comparison of the following: (a) adherence to the EMI; (b) changes in the perceived helpfulness and difficulty of intervention over the course of the study; (c) changes in the mental health indices (i.e. depressive and anxiety symptoms as well as perceived stress in baseline versus follow-up assessment); and (d) self-reported target engagement (i.e. changes in the tendency to use PCR in baseline versus follow-up assessment) between the burst and continuous intervention delivery designs of a mobile app employing a core CBT technique and the important resilience factor - positive cognitive reappraisal.

Despite the BD group not receiving reminders to use the app for 1 week during the study, there was no statistically significant difference in the overall adherence rate (i.e. the number of completed surveys) between the groups. There were no significant differences between the groups in any of the mental health outcomes (i.e. perceived stress, anxiety symptoms, and depressive symptoms) as well as in the two scales measuring the tendency to use PCR. There was a significant difference in the perceived difficulty of the intervention, meaning that the PCR generation became significantly easier over time for the BD group, whereas no such change was observed for the CD group. Compared to the BD group, the CD group consistently assessed the intervention as more helpful, irrespectively of time. The correlation analysis revealed a modest association between the reappraisals which were easier for participants to generate, and their higher perceived helpfulness, especially in the BD group.

These findings suggest that the burst design may be just as effective as the continuous design for digital mental health interventions supporting the mental well-being, at least for the reappraisal-based EMIs. Across most of the measured outcomes, there were either no significant differences between the groups or only marginal disparities in effect sizes, but with reduced participant burden in the burst delivery group. The sole significant difference observed over the course of the study was in the perceived difficulty of the intervention, which showed a notable decrease only in the BD group.

One of the putative mechanisms induced by the burst delivery of the digital interventions may be the sense of agency in the intervention process. The sense of agency refers to the awareness that individuals have control over their actions and thoughts^38^ and the consequences of these actions.^39^ Allowing the BD group participants to self-initiate the intervention without reminders in the second week of the study may have enhanced their sense of agency. Enhancing the client's agency is indeed a key concept in psychotherapy, and many psychotherapeutic protocols define the improvement in the mental well-being of the client as a result of mobilizing their agency and using interventions to heal themselves.^40^ Empirical evidence suggests that increases in the sense of agency during the therapeutic process are related to improvements in mental health outcomes.^41^ However, little is known about the role of the sense of agency in digital mental health interventions. Existing studies suggest that mHealth EMIs indeed provide a sense of agency to boost an active role in managing depression, anxiety, and somatoform disorders in clinical populations^15^ and increase the sense of agency after discharge from the hospital.^42^ However, research on preventative EMIs is scarce; hence, we cannot directly pinpoint the changes in the BD outcomes to this mechanism.

Another explanation may be that participants who were less burdened with the app were more internally motivated to be involved in the process, thereby performing better in the PCR component and achieving somewhat better outcomes in terms of the perceived difficulty of the intervention. In contrast, the continuous reminders in the CD group may have influenced external motivation, possibly leading to a habitual completion of the PCR component without genuine engagement. Previous reports suggested that low treatment motivation predicts the dropout rate and therapeutic success in anxiety disorders.^43^ Participants with anxiety symptoms may possibly respond better to continuous reminders about the treatment than to the burst delivery of interventions whose use is highly dependent on the participants’ motivation to continue the process. Accordingly, future research could investigate the individual differences in the response patterns concerning the two delivery designs. This could be a key to finding the most suitable delivery approach tailored to individuals facing distinct mental health challenges.

This study has a few limitations, including a homogenous, mostly female sample with a low tendency to use PCR, warranting replications in diverse general populations and clinical samples. We have not only observed an increased interest in the interventional study in female participants, which is a common case in psychological research, but also a higher tendency to use PCR, as indexed with CERQ, in men. As a result, many male participants have not fulfilled the inclusion criteria of this study. The study design could be improved by introducing longer intervention periods with alternating burst and “quiet” periods to assess the stability of the burst delivery effects. Additionally, more research is needed to validate the use of burst delivery in digital interventions targeting alternative domains and therapeutic techniques.

## Conclusion

The current study showed that the burst delivery design holds promise for maintaining the effectiveness of digital mental health interventions while alleviating participant burden, thereby offering a potential improvement over the continuous delivery method. Although this addresses one of the major challenges in the mHealth field, more studies are needed to find a mechanism underlying these differences. Nonetheless, our findings can inform future research in terms of designing digital mental health interventions as well as feasibility studies and randomized clinical trials with less participant effort.

## Supplemental Material

sj-docx-1-dhj-10.1177_20552076241249267 - Supplemental material for Burst versus continuous delivery design in digital mental health interventions: Evidence from a randomized clinical trial Supplemental material, sj-docx-1-dhj-10.1177_20552076241249267 for Burst versus continuous delivery design in digital mental health interventions: Evidence from a randomized clinical trial by Marta Anna Marciniak, Lilly Shanahan, Kenneth S L Yuen, Ilya Milos Veer, Henrik Walter, Oliver Tuescher, Dorota Kobylińska, Raffael Kalisch, Erno Hermans, Harald Binder and Birgit Kleim in DIGITAL HEALTH

sj-doc-2-dhj-10.1177_20552076241249267 - Supplemental material for Burst versus continuous delivery design in digital mental health interventions: Evidence from a randomized clinical trial Supplemental material, sj-doc-2-dhj-10.1177_20552076241249267 for Burst versus continuous delivery design in digital mental health interventions: Evidence from a randomized clinical trial by Marta Anna Marciniak, Lilly Shanahan, Kenneth S L Yuen, Ilya Milos Veer, Henrik Walter, Oliver Tuescher, Dorota Kobylińska, Raffael Kalisch, Erno Hermans, Harald Binder and Birgit Kleim in DIGITAL HEALTH
