# Supplementary material for: Burst versus continuous delivery design in digital mental health interventions: Evidence from a randomized clinical trial
Source: Digit Health. 2024 Apr 30;10:20552076241249267. doi: 10.1177/20552076241249267 (PMC11064753; doi:10.1177/20552076241249267)
Supplement: sj-docx-1-dhj-10.1177_20552076241249267 - Supplemental material for Burst versus continuous delivery design in digital mental health interventions: Evidence from a randomized clinical trial [file sj-docx-1-dhj-10.1177_20552076241249267.docx]

**Supplement 1.**

Conditional R2 for each of the models

| **Outcome** | **Value marginal (R2)** |
| --- | --- |
| **Perceived helpfulness**  Time  Group  Interaction | 0.000  **0.026**  **0.026** |
| **Perceived difficulty**  Time  Group  Interaction | 0.002  0.001  **0.006** |
| **Perceived stress (PSS)**  Time  Group  Interaction | **0.065**  0.001  **0.065** |
| **Anxiety symptoms (STAI)**  Time  Group  Interaction | 0.011  0.009  **0.021** |
| **Depressive symptoms (BDI-II)**  Time  Group  Interaction | 0.074  0.000  **0.076** |
| **Reappraisal (CERQ)**  Time  Group  Interaction | 0.258  0.006  **0.262** |
| **Reappraisal (ERQ)**  Time  Group  Interaction | 0.064  0.103  **0.138** |
